# Small RNA Profiles of Serum-Derived Extracellular Vesicles in the Comorbid Condition of Frailty and Obstructive Pulmonary Disease: An Observational, Cross-Sectional Study

**DOI:** 10.3390/biom15121663

**Published:** 2025-11-28

**Authors:** Keiko Doi, Tsunahiko Hirano, Naoomi Tominaga, Kenji Watanabe, Keiji Oishi, Ayumi Fukatsu-Chikumoto, Tasuku Yamamoto, Yuichi Ohteru, Kazuki Hamada, Yoriyuki Murata, Maki Asami-Noyama, Nobutaka Edakuni, Tomoyuki Kakugawa, Yoichi Mizukami, Kazuto Matsunaga

**Affiliations:** 1 Department of Respiratory Medicine and Infectious Disease, Graduate School of Medicine, Yamaguchi University, 1-1-1 Minami-Kogushi, Ube 755-8505, Japan; decem119@yamaguchi-u.ac.jp (K.D.); khamada@yamaguchi-u.ac.jp (K.H.);; 2 Department of Pulmonology and Gerontology, Graduate School of Medicine, Yamaguchi University, 1-1-1 Minami-Kogushi, Ube 755-8505, Japan; kakugawa@yamaguchi-u.ac.jp; 3 Division of Clinical Laboratory Sciences, Department of Nursing and Laboratory Science, Graduate School of Medicine, Yamaguchi University, 1-1-1 Minami-Kogushi, Ube 755-8505, Japan; 4 Institute of Gene Research, Yamaguchi University Science Research Center, 1-1-1 Minami-Kogushi, Ube 755-8505, Japan

**Keywords:** extracellular vesicles, microRNAs, RNA, small non-coding, muscle strength, exercise test, aging

## Abstract

Frailty is increasingly recognized as a systemic complication in patients with obstructive pulmonary diseases (OPDs), yet its molecular basis remains unclear. Extracellular vesicles (EVs), which transport small RNAs, may offer mechanistic insight into this comorbidity. This study investigated the association between serum-derived EV small RNAs and frailty in OPD. Sixty-eight patients with OPD were enrolled, and EVs isolated from 29 patients (13 with chronic obstructive pulmonary disease [COPD], four with COPD and asthma, and 12 with asthma; median age 72 years) were analyzed. Based on the Kihon Checklist, patients were classified as frail (*n* = 11) or non-frail (*n* = 18). Small RNA sequencing and differential expression analyses were conducted, followed by age-adjusted correlation with physical factors and Ingenuity Pathway Analysis (IPA). A total of 108 small RNAs were differentially expressed between frail and non-frail groups (*p* < 0.05, fold change < 0.8 or >1.2). IPA linked these RNAs to lung fibrosis and transforming growth factor-beta (TGF-β) signaling pathways. Eleven small RNAs correlated with lower limb strength, and three—miR-125b-5p, miR-369-3p, and miR-615-3p—emerged as key candidates associated with frailty. These findings suggest that EV-derived small RNAs may contribute to frailty development in OPD through TGF-β–related molecular mechanisms.

## 1. Introduction

As the older population increases, chronic respiratory diseases, particularly obstructive pulmonary disease, are also increasing [1,2]. These conditions cause shortness of breath (dyspnea), reducing physical activity and leading to muscle weakness and loss of muscle mass. In patients with COPD, frailty and sedentary lifestyles have also been associated with cognitive decline [3]. This functional decline has been linked to poorer quality of life and prognosis, posing significant social challenges [1]. A major contributing factor is the presence of frailty [3,4,5,6,7,8,9].

Frailty, a syndrome marked by reduced physiological reserve related to aging, increases vulnerability to stressors, potentially leading to functional impairment, dependency, and increased mortality [10]. The pathophysiology of frailty is closely associated with chronic inflammation, immune activation, musculoskeletal dysregulation, oxidative stress, and mitochondrial dysfunction [11,12,13]. However, the mechanisms underlying the comorbidity of frailty and obstructive pulmonary disease remain complex and not fully understood.

Recent research has focused on extracellular vesicles (EVs), which play crucial roles in intercellular communication. EVs contain proteins, lipids, and nucleic acids such as messenger RNA (mRNA), microRNA (miRNA), PIWI-interacting RNA (piRNA), and DNA. These molecules are released into the extracellular space and delivered to recipient cells, facilitating communication across distant organs and tissues [14,15,16]. Small RNAs, including miRNAs and piRNAs, regulate gene expression primarily at the post-transcriptional stage [17]. miRNAs in particular influence numerous biological processes, including development, cell differentiation, cancer, and stress responses. Therefore, EV small RNA signatures are useful biomarkers and tools for investigating molecular mechanisms of disease [18,19]. Moreover, EVs transport bioactive substances that contribute to the progression of muscle wasting [20,21]. In this context, we hypothesized that small RNAs in serum-derived EVs play a significant role in mediating the comorbid state of frailty and obstructive pulmonary disease. This study aimed to elucidate the role of serum-derived EV small RNAs in this relationship.

## 2. Materials and Methods

### 2.1. Study Patients

We recruited patients with pulmonary diseases undergoing treatment at the Department of Respiratory and Infectious Diseases, Yamaguchi University Hospital, from June 2021 to July 2022. The patients were older than 20 years. The study protocol and amendments were approved by the institutional review board of Yamaguchi University (approval no. 2021-031). Written informed consent was obtained from all patients. Only patients with available serum samples were included in the analysis.

The disease type, sex, age, and smoking history of patients were obtained through interviews. A pulmonologist confirmed the diagnoses of COPD or asthma. Patients were clinically stable and had no exacerbations for at least 4 weeks prior to the study. No participant had injuries or leg paralysis.

### 2.2. Measurements

#### 2.2.1. Frailty Status

Frailty status was classified into three groups (frail, pre-frail, and robust) using the Kihon Checklist (KCL) [22]. KCL is a 25-item questionnaire that evaluates instrumental and social activities of daily living, physical function, nutritional status, oral function, cognitive function, and depressive mood. It was developed by the Ministry of Health, Labor, and Welfare of Japan for nursing care prevention. Owing to limitations in sample size, we combined robust and pre-frail into a single non-frail category. If the score was 0–7, the participant was categorized as non-frail, and 8–25 as frail.

#### 2.2.2. Pulmonary Function Assessment

Pulmonary function was assessed according to the recommendations of the American Thoracic Society and European Respiratory Society [23], using a multifunctional spirometer (HI-801, Chest Ltd., Tokyo, Japan). Japanese reference values were used [24].

#### 2.2.3. 6 Min Walk Test (6MWT)

The 6MWT was performed according to the American Thoracic Society Guidelines [25]. Patients were instructed to walk as far as possible along a flat, straight surface marked at 1 m intervals. The total walking distance was visually measured, and walking speed was calculated by dividing distance by walking time.

#### 2.2.4. Handgrip Strength (HS)

HS was measured using a digital grip dynamometer (TAKEI Scientific Instruments Co., Ltd., Niigata, Japan). Patients were instructed to stand upright, allow their arms to hang naturally, and grasp the device with full force. Two readings were obtained from each hand, and the higher score was recorded as the HS value [26].

#### 2.2.5. Lower Limb Strength (LLS)

Lower limb strength was measured using a handheld dynamometer (μTas F-1; Anima Corp., Tokyo, Japan) in a sitting position, with a belt attached to the bedpost and ankle. Patients were positioned with the hip and knee joints at 90° flexion and instructed to extend the knee joint with maximal effort. Measurements were taken three times on each side [27].

#### 2.2.6. Body Compositions

Body composition parameters such as skeletal muscle index (SMI) and whole-body phase angle (WBPhA) were measured by bioelectrical impedance analysis [28] using InBody S10 (InBody Japan Inc., Tokyo, Japan) in the supine position. SMI was calculated by dividing the mass of the limb skeletal muscle (kg) by the square of the height (m^2^).

### 2.3. Serum-Derived EV Isolation

#### 2.3.1. Blood Collection and Processing

Peripheral blood samples were collected from 68 patients into Venoject II vacuum tubes containing a gel for serum separation (maximum volume per tube = 8 mL). After incubation at room temperature (RT) and centrifugation, the serum layer was aliquoted and stored at −80 °C until EV isolation. After thawing, the serum samples were centrifuged at 1500× *g* for 10 min at RT to remove cells and large particles, followed by re-centrifugation at 10,000× *g* for 10 min at RT.

#### 2.3.2. Size Exclusion Chromatography (SEC) EV Isolation

Serum samples (0.5 mL) were fractionated through SEC with the qEV column (IZON, Cat Gen2 qEV original 70 nm). The column was flushed with 8.5 mL of 0.2 μm filtered phosphate-buffered saline (PBS) prior to sample loading. Samples were loaded and run with 8 mL of PBS, and the first 1.5 mL was pooled. Ten fractions (0.5 mL each) were then collected up to 5 mL. The column was regenerated with 8.5 mL of 0.5 M sodium hydroxide (NaOH) followed by 17 mL of buffer before reuse. Columns were used up to 5 times as instructed. EVs were stored at 4 °C. Because the method did not exclude the possibility of contamination from other EV subgroups, such as microvesicles, we utilize the term EVs to refer to the vesicles collected in this procedure.

#### 2.3.3. Nanoparticle Tracking Analysis (NTA) of EVs

Videodrop (Myriade, Paris, France) was used to determine the size distribution of the EVs. All samples were checked.

#### 2.3.4. Protein Determination in EV Fractions

After the SEC, protein content in each EV fraction was determined using the Micro BCA Protein Assay Reagent Kit (Thermo Fisher Scientific, Rockford, IL, USA) following the manufacturer’s instructions. We measured the first four samples.

#### 2.3.5. Western Blot of EV Markers

Each EV fraction sample were mixed with Laemmli sample buffer (Bio-Rad, Hercules, CA, USA) under non-reducing conditions and incubated for 30 min at 37 °C. Proteins were separated by SDS-polyacrylamide gel electrophoresis (SDS-PAGE) and transferred onto polyvinylidene difluoride (PVDF) membranes. Membranes were blocked with Every Blot Blocking Buffer (Bio-Rad, USA) for 60 min at RT and incubated for 60 min at RT with Anti-CD63 Monoclonal Antibody (3-13), diluted 1:1000 (Cat 012-27063, Fujifilm, Tokyo, Japan). Following three washes with tris-buffered saline containing 0.1% Tween 20 (TBS-T), membranes were incubated with secondary antibody, goat anti-mouse IgG (H + L) horseradish peroxidase -conjugate (1:3000; Cat 170-6516, Bio-Rad, Hercules, CA, USA) for 60 min at RT. Following 3 additional washes with TBS-T, membranes were visualized using Clarity Western enhanced chemiluminescence (ECL) substrate (Bio-Rad, Hercules, CA, USA). Amersham Imager 600 (Cytiva, Marlborough, MA, USA) was used to perform imaging. All samples were checked.

#### 2.3.6. Transmission Electron Microscopy (TEM)

A formvar/carbon coated copper grid (Cat#6515, New EM Co., Ltd., Tokyo, Japan) was hydrophilized using a JFC-1600 Auto Fine Coater (JEOL Ltd., Tokyo, Japan). Purified EVs (3 μL in PBS) were applied onto the hydrophilized grids and adsorbed for 3 min. The grid was washed with 500 μL double-distilled H_2_O four successive drops, and subsequently negatively stained with 30 μL of 2.0% uranyl acetate successive 4 drops. The grid was air-dried after excess stain was removed with filter paper. Imaging was performed using a Tecnai G2 Spirit BioTWIN electron microscope (FEI Company, Hillsboro, OR, USA) operating at 120 kV, equipped with a Phurona CMOS camera (Emsis Münster, Germany). Raw images were exported in TIFF format and analyzed using Fiji (ImageJ 1.53t).

### 2.4. RNA Extraction and Next Generation Sequence (NGS)

#### 2.4.1. Total RNA Extraction from EVs

EVs obtained from serum were stored in PBS at 4 °C; RNA was isolated using the miRNeasy Serum/Plasma Advanced Kit (Cat 217204, QIAGEN, Hilden, Germany), following the manufacturer’s instructions.

#### 2.4.2. Library Preparation and RNA-Seq

A QIAseq^®^ miRNA Library Kit (QIAGEN, Hilden, Germany) was used to generate the library, according to the manufacturer’s instructions. Agilent 2200 TapeStation (D1000, Agilent Technologies, Inc., Santa Clara, CA, USA) was used to confirm the quality of the library. Paired-end sequencing at 100 bp was performed on a NovaSeq 6000 (Illumina, San Diego, CA, USA) using a Novaseq 6000 S1 Reagent kit. Primary analysis was performed using the RNA-seq Analysis portal (geneglobe.qiagen.com, 25 October 2024), where unique molecular identifiers were counted, and miRNA sequences were mapped. Data were normalized to counts per million (CPM) and converted to log_2_ (CPM + 1).

#### 2.4.3. Ingenuity Pathway Analysis (IPA)

Small RNA-seq data were analyzed using IPA (QIAGEN GmbH, Hilden, Germany) to identify pathogenetic pathways contributing to frailty mechanisms in obstructive pulmonary disease. Genes with *p* < 0.05 and FC < 0.8 or FC > 1.2 were included. The enrichment ratio (circle size) was based on the *p*-value and number of molecules in the Diseases and Functions analysis of IPA.

### 2.5. Statistical Analysis

Differential small RNA expression was calculated using an unpaired Student’s *t*-test in Microsoft Excel to generate the volcano plot and visualize potentially promising miRNAs. Group differences were assessed using the Wilcoxon/Kruskal–Wallis test, and categorical data were analyzed using Pearson’s chi-squared (χ^2^) test. Correlations between physical factors and small RNAs were analyzed using Spearman’s rank correlation coefficients and partial correlations. Statistical analyses and volcano plots were performed using JMP Pro^®^, version 18 (SAS Institute Inc., Cary, NC, USA). Statistical significance was defined as a *p* < 0.05. Heat maps were created using the Morpheus web server (https://software.broadinstitute.org/morpheus/, 16 October 2024). False discovery rate (FDR) adjustment was performed using GraphPad Prism 10.6.1, (GraphPad Software Inc., Boston, MA, USA).

## 3. Results

### 3.1. Characteristics of the Study Population

The participant characteristics are summarized in Table 1 (flowchart, Figure S1). The patients comprised 23 males and six females, with a median age of 72 years. Eleven were categorized as frail and 18 as non-frail. Contrary to expectations, there were no significant differences in SMI, HS, disease history, or smoking history between groups. However, LLS and 6MWT values were significantly lower in the frail group (*p* = 0.015, *p* = 0.010, and *p* = 0.029). Additionally, the non-frail group exhibited a significantly lower median age (*p* = 0.041).

### 3.2. Serum EV Characteristics

**Figure 1:**
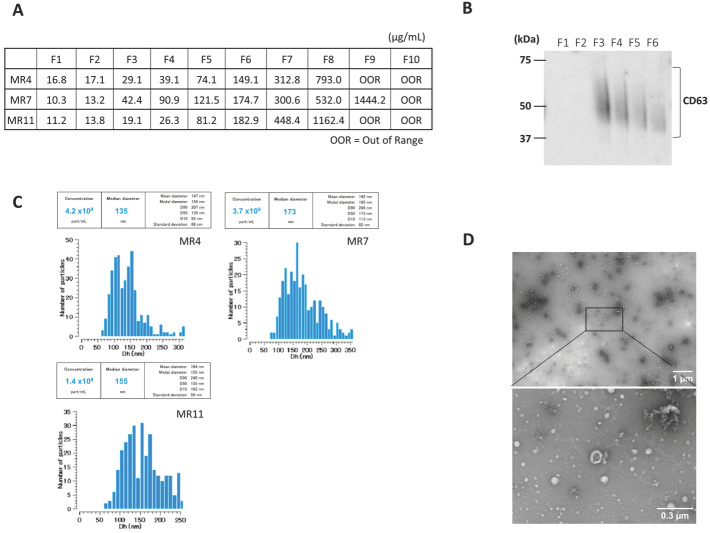
Characterization of serum-derived small extracellular vesicles (EVs). A representative sample is presented here. (**A**) Protein Concentration in each fraction of SEC. (**B**) CD63 expression in each fraction of SEC was determined by Western blot (original image can be found in Figure S2). (**C**) Representative particle size distribution profile of nanoparticle tracking analysis (NTA) of EVs from serum. (**D**) Representative transmission electron microscopy (TEM) images of EVs isolated by SEC. Scale bars are 1 um and 300 nm on the right side of the images.

Serum-derived EVs were characterized after SEC. The protein concentration increased with fraction number (Figure 1A). Next, we confirmed the presence of CD63, an exosome marker, in fraction 3–6, using Western blot (Figure 1B). Videodrop (Myriade, France) showed EV size distribution of approximately 90–240 nm, peaking at ~150 nm (Figure 1C). TEM confirmed intact vesicles with the typical round, cup-shaped morphology (~200 nm) (Figure 1D). These results suggest that EVs were successfully isolated.

### 3.3. Small RNA Characteristics from EVs

**Figure 2:**
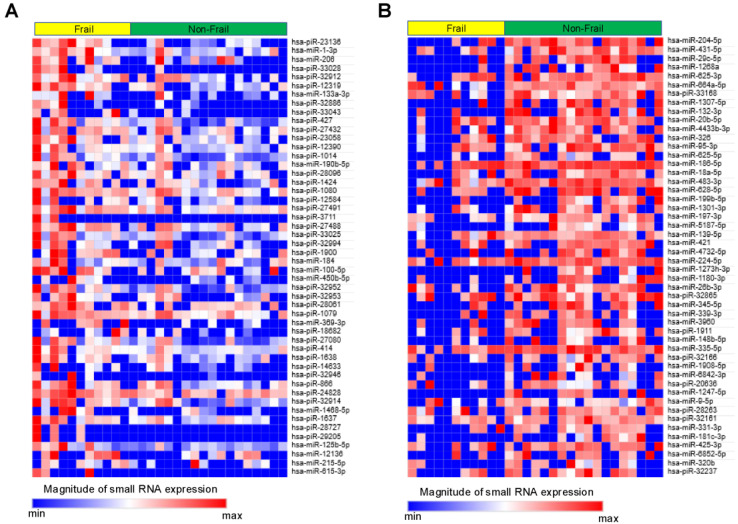
Heat map showing relative expression levels of small RNAs in the Frail vs. Non-Frail groups. (**A**) Top 50 small RNAs upregulated in Frail vs. Non-Frail groups. (**B**) Top 50 small RNAs downregulated in Frail vs. Non-Frail groups. A relative color scheme uses the minimum and maximum values in each row to convert values into colors.

**Figure 3:**
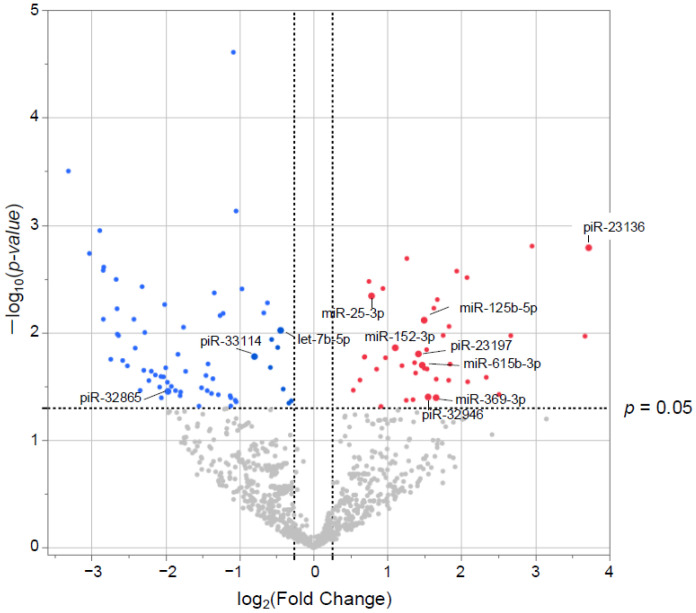
Differentially expressed (DE) small RNAs in volcano plots. The *x*-axis represents the log2 conversion of the fold-change values, whereas the *y*-axis represents the significance level after log10 conversion. Blue (downregulated) and red (upregulated) dots indicate small RNAs with |log2 fold change| > 0.26 (vertical dashed lines) and *p* < 0.05 (horizontal dashed line).

Small RNAs from EVs of 29 patients underwent NGS. Of the 1015 small RNAs detected, heat maps showed the top 50 differentially expressed small RNAs from frail vs. non-frail groups (Figure 2A,B, and Table S1A,B). Forty-two were upregulated and 66 downregulated with |log_2_ FC| > 0.26 and unadjusted *p* < 0.05 (Figure 3). The 10 most significant small RNAs included miR-126-3p, miR-204-5p, miR-150-5p, miR-29c-5p, piR-33028, piR-23136, miR-431-5p, miR-451a, miR-664a-5p, and miR-1268a (Table S2). No significant differences were found after a false discovery rate (FDR) adjustment, except for miR-126-3p (*q* < 0.05) (Table S2).

### 3.4. Correlation Between Small RNA and Physical Factors

As frailty and physical function are closely related, we examined the correlation between significantly differentially expressed small RNAs and physical factors. First, Spearman rank correlations were calculated between Left LLS (Lt LLS), right LLS (Rt LLS), the 6MWT, and the top 100 differentially expressed small RNAs. From this, 16 small RNAs significantly correlated with Lt LLS, which had the most significant difference between the frail and non-frail groups. Among these, piR-23136 had the strongest negative correlation with Rt LLS (r = −0.61, *p* < 0.01), Lt LLS (r = −0.58, *p* < 0.01), and the 6MWT (r = −0.54, *p* < 0.01) (Table S3). Partial correlations adjusted for age (Table 2) showed 11 small RNAs—including miR-369-3p, piR-23136, piR-33114, piR-23197, piR-32865, miR-125b-5p, let-7b-5p, miR-25-3p, miR-152-3p, piR-32946, and miR-615-3p—remained significantly correlated with Lt and Rt LLS.

### 3.5. Ingenuity Pathway Analysis (IPA)

**Figure 4:**
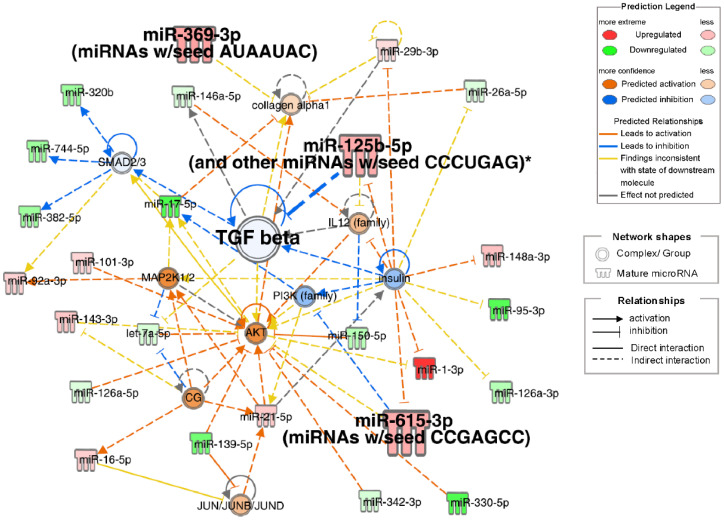
Network diagrams with high IPA scores. Ingenuity pathway analysis (IPA) of differentially expressed miRNAs in Frail compared to non-frail groups. Top network associated with TGF beta, insulin, and collagen alpha1 signals. Node Colors: Red indicates molecules that are upregulated, with darker red representing more extreme upregulation. Green denotes downregulated molecules, with darker green representing more extreme downregulation. Orange represents the predicted activation of a molecule or process, with darker shades indicating higher confidence. Blue indicates the predicted inhibition, with darker shades representing higher confidence. Edge Types (Predicted Relationships): Orange lines signify the relationships that lead to activation. The blue lines signify the relationships that lead to inhibition. Yellow lines represent findings inconsistent with the predicted state of the downstream molecule. Gray lines indicate relationships in which the effect was not predicted. *: Duplicates-Gene/Protein/Chemical identifiers marked with an asterisk indicate that multiple identifiers in the dataset file map to a single gene/chemical in the Global Molecular Network.

IPA of small RNA-seq data identified pathogenetic pathways contributing to frailty in patients with obstructive pulmonary disease. The highest-scoring network included TGF-β, collagen alpha-1, insulin, AKT, IL12, MAP2K1/2, PI3K, and SMAD2/3-related signals (Figure 4). Insulin downregulation was associated with the downregulation of TGF-β and PI3K, with upregulation of IL12, AKT, miR-125b-5p, miR-615-3p, and miR-1-3p. miR-125b-5p upregulation inhibited TGF-β, which correlated with collagen alpha-1 upregulation. Small RNAs significantly correlated with physical factors (miR-125b-5p, miR-369-3p, and miR-615-3p) were involved in a top network that included TGF beta. Other than network analysis, top 20 analysis-ready molecules (upregulated top 10 and downregulated top 10) were selected (Table S4).

### 3.6. Identification of Candidate Small RNAs

**Figure 5:**
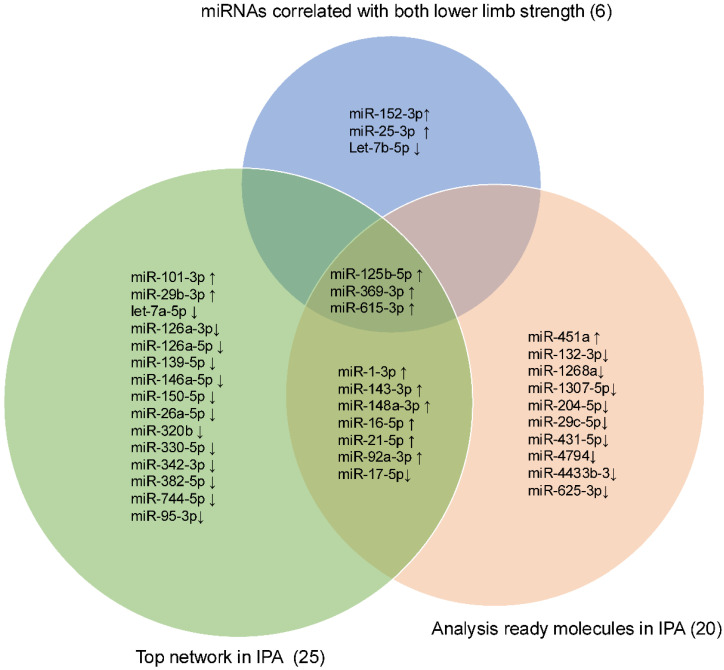
Venn diagram showing the intersection among the lower limb strength, the Top network, and the Analysis Ready Molecules in IPA. Venn diagram showing the intersection of three candidate miRNAs: miR-125b-5p, miR-369-3p, and miR-615-3p. The upward arrow indicates upregulation, and the downward arrow indicates downregulation in frail.

**Figure 6:**
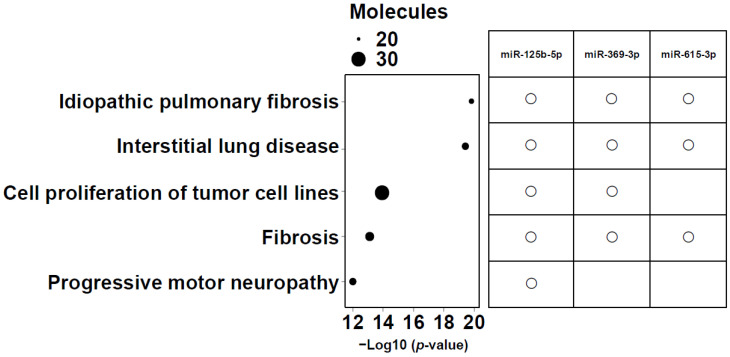
Enrichment ratio (size of the circle) and *p*-values for the 5 most enriched diseases and functions. The top five diseases and biofunctions that met the conditions of −log10 (*p* value) > 11, and molecules > 20 are shown. The size of the bubbles corresponds to the number of miRNAs involved in each category. The right panel shows a matrix indicating which candidate microRNAs (miR-125b-5p, miR-369-3p, and miR-615-3p) are associated with each of the listed diseases or biofunctions.

Intersection operations were conducted among 6 miRNAs correlated with lower LLS, 25 miRNAs in the network, and the top 20 analysis-ready molecules in IPA. The overlapping small RNAs in these groups were miR-125b-5p, miR-369-3p, and miR-615-3p (Figure 5). These three miRNAs were significantly highly expressed in frail individuals (Figure S3). Figure 6 shows the enrichment ratio (circle size). Idiopathic pulmonary fibrosis, interstitial lung disease, and fibrosis were associated with dysregulation of miR-125b-5p, miR-369-3p, and miR-615-3p. For piRNAs, network analysis was not possible because of the large number of molecules with unknown functions and targets, as well as the low accuracy of target prediction. Nevertheless, we selected piR-23136 (*p* < 0.01) and piR-33114 (*p* < 0.017) as candidates, as they differed significantly between frail and non-frail groups and correlated with lower LLS and 6MWT (Figure S4, Table 2). For candidate small RNAs, we performed logistic regression analyses adjusted for age and gender (Table S5). Among miR-125b-5p, miR-615b-3p, miR-369b-3p, piR-23136, and piR-33114, only miR-369b-3p lost statistical significance after adjustment.

## 4. Discussion

Specific small RNAs—miR-125b-5p, miR-369-3p, and miR-615-3p—carried by serum-derived EVs were identified as potential mediators of frailty-related comorbidities in patients with obstructive pulmonary diseases. These three miRNAs were associated with LLS and were involved in the network analysis, including TGF-β.

To our knowledge, this is the first study to perform NGS analysis of EV small RNAs from 11 frail and 18 non-frail individuals with obstructive respiratory disease. Analyses of small RNAs from EVs fractionated by SEC and confirmed for CD63 expression have not been reported in other frailty studies.

Profiling miRNAs derived from EVs isolated from plasma identified eight miRNAs (miR-10a-3p, miR-92a-3p, miR-185-3p, miR-194-5p, miR-326, miR-532-5p, miR-576-5p, and miR-760) specifically enriched in frailty [29]. Based on NGS, only miR-92a-3p and miR-326 were consistent with the present results. miR-92a-3p has been extensively studied and is known to enhance interleukin-6 production. KEGG analysis identified some pathways involved in aging, including insulin, AMPK, and FoxO signaling, among the top 50 pathways, but no lung disease-related pathways. These differences may be due to the EV isolation approach and study participants, which differed from ours. Their samples were derived from plasma, and participants did not have chronic lung disease and included young healthy adults (23–35 years old). The two overlapping miRNAs may be markers for frailty in general, but not specific to frail patients with respiratory diseases.

Hannah E. O’Farrell et al. reported that plasma EV miRNAs hold biological information regarding the severity of airflow obstruction and COPD exacerbations [30]. Similarly, serum exosomal miR-1258 is associated with inflammation and could serve as a reliable biomarker for diagnosing acute COPD exacerbations [31]. These findings indicate that EV miRNA abundance in blood changes with the pathology of obstructive pulmonary disease. However, reports of EV miRNAs from blood samples of such patients showed little overlap with the small RNAs identified here.

Another study treated old mice with EVs from adipose mesenchymal stem cells (ADSCs) of young animals and found improvement in frailty parameters [32]. Among the 6 EV-derived miRNAs identified as significant in improving frailty in that study, miR-125b-5p and miR-143-3p were also significant here; however, the regulation direction differed, possibly because of species differences and whether EVs were cell- or serum-derived. However, these two miRNAs may be involved in controlling frailty symptoms.

Several miRNAs are differentially expressed in frail serum and plasma. A recent meta-analysis identified miR-125b-5p as a protective biomarker in frailty [33]. Although that study analyzed serum miRNAs using qPCR, it is notable that miR-125b-5p emerged as a biomarker among 35,000 omics and routine laboratory variables.

In the IPA, the top diseases and disorders were cancer, organismal injury and abnormalities, reproductive system disease, gastrointestinal disease, and respiratory disease, many of which were associated with aging. The top physiological system development and function categories were skeletal and muscular system development and function, tissue morphology, embryonic development, and connective tissue development and function. These findings indicate changes in skeletal, muscular, and tissue development in frailty. In the network analysis (Figure 4), the three candidate miRNAs—miR-125b-5p, miR-369-3p, and miR-615-3p—selected from the Venn diagram (Figure 5) were involved in TGF-β signaling. miR-125b-5p elevation was linked to TGF-β inhibition; miR-369-3p regulated collagen alpha 1 with TGF-β, and miR-615-3p regulated AKT with TGF-β.

TGF-β plays a crucial role in airway remodeling in asthma and COPD. By acting on epithelial cells, fibroblasts, smooth muscle cells, and immune cells, it promotes extracellular matrix production, smooth muscle hypertrophy, and fibrosis, leading to airway wall thickening and irreversible airflow limitation. Pro- and anti-inflammatory functions of TGF-β have been reported in these diseases. Figure 6 reflects this, with idiopathic pulmonary fibrosis, interstitial lung disease, and fibrosis ranked as the most related diseases.

Previous studies have associated miR-125b-5p with the regulation of vitamin D receptor (VDR) expression [34]. VDR exists in almost all tissues and regulates cell proliferation, differentiation, immunomodulation, and skeletal muscle function [19]. Vitamin D deficiency has been associated with acute lung injury, pneumonia, asthma, tuberculosis, and COPD [35]. Additionally, miR-125b-5p (miR-125) is upregulated in the brain tissue, cerebrospinal fluid, and serum EVs of patients with Alzheimer’s disease [36]. Thus, miR-125b-5p may play a major role in pulmonary disease and frailty associated with inflammation, cognitive decline, and skeletal muscle dysfunction. Numerous studies have reported the role of miR-369-3p in regulating inflammation [37,38,39]. miR-369-3p overexpression may reduce pulmonary edema, inflammation, and permeability in mice with LPS-induced acute lung injury [40]. Liu et al. showed that miR-615-3p directly binds to the 3′-UTR of IGF2 in vitro [41]. IGF2 may be involved in memory and its regeneration [42,43]. IGF2 deficiency has been associated with neurodegenerative diseases such as Alzheimer’s, Parkinson’s, and Huntington’s diseases [43], possibly contributing to cognitive decline and muscle weakness in frail patients. These three molecules were involved in a network that includes TGF-β signaling (Figure 4). TargetScan predicted that miR-125b-5p and miR-615-3p target TGFBR1 (TGF-β receptor 1), suggesting that they may regulate lung fibrosis through TGF-β.

Here, piRNAs were among the molecules upregulated in frailty (Figure 2 and Figure S4, and Table S1A). piRNAs are small non-coding RNAs (24–32 nucleotides) representing the largest class of small non-coding RNAs in animal cells. They are expressed in various human tissues and influence critical signaling pathways at transcriptional and post-transcriptional levels [44,45,46]. Additionally, altered expression of piRNAs and PIWI proteins has been observed in several cancers [47,48]. hsa-piR-23136, which showed the highest upregulation by FC (Figure 2A, Table S1A), ranked sixth with a significant difference (Figure 3, Table S2) in the frail group and was also significantly correlated with physical factors (Table 2). However, its target remains unclear, and there have only been reports of its downregulation in patients with acute ischemic stroke [49]. Future research should focus on elucidating the role of EV-derived piRNAs in frailty development in obstructive pulmonary disease.

Interestingly, previously, we found that serum GDF-15 was associated with physical inactivity and cognitive decline in a similar patient population [50]. Although the precise mechanisms remain unclear, these observations suggest that circulating factors—whether blood molecules such as GDF-15 or EV-derived small RNAs—may mediate the link between pulmonary pathology and systemic frailty. A decline in LLS may indirectly lead to an increase in miR-125b-5p, miR-369-3p, and miR-615-3p in blood EVs, which are then transported to the lungs and bronchi. This process might promote pathological conditions such as inflammation and airway narrowing in obstructive diseases through changes in TGF-β signaling. This TGF-β-related molecular mechanism contributes to frailty development in COPD which is informative findings for biomarker discovery as well as drug discovery. Further research, including in vitro and in vivo experiments, is needed to verify the interplay between these small RNAs and the pathophysiology of frailty in obstructive pulmonary diseases.

### Limitations

This study had several limitations. It was conducted at a single institution, limiting the sample size and restricting patients to one ethnic group. There were no frail participants without obstructive pulmonary disease. Some samples did not yield enough small RNA for NGS. Additionally, due to the very low amount of EV-derived small RNA, qPCR validation was not possible. We plan to validate candidate small RNAs in future studies and investigate their effects in cells. The small number of females and the significant age difference between frail and non-frail individuals may have influenced the results. After adjusting for age and gender, most candidate small RNAs remained significant, except miR-369b-3p (Table S5). Because the number of frail females was extremely limited (1 out of 6), the statistical power to assess gender-specific effects was insufficient. Although miR-125b-5p has been discussed in relation to aging [51,52,53], no clear age-related confounding was observed in our EV-derived data. Few studies have examined age-related aspects of serum EV-derived miR-125b-5p, and it is possible that this is influenced not by age itself but by age-related conditions such as frailty. Although molecular candidates linking obstructive pulmonary diseases and frailty have been identified, the cross-sectional design prevents the determination of causality. Further studies, including longitudinal investigations in larger populations with diverse ethnic backgrounds, are needed to confirm their clinical relevance.

## 5. Conclusions

Small RNAs carried by serum-derived EVs may play a crucial role in mediating the coexistence of frailty in obstructive pulmonary diseases, particularly through involvement in the TGF-β signaling pathway. Further studies are required to elucidate the underlying mechanisms and assess their potential as biomarkers.

## Figures and Tables

**Table 1:** Characteristics of the study patients.

Characteristics	All (*n* = 29)	Frail (*n* = 11)	Non-Frail (*n* = 18)	*p*-Value
Age (years)	72.0 (65.5–77.0)	75.0 (69.0–82.0)	70.0 (63.8–73.8)	0.041
Gender (male/female)	23/6	10/1	13/5	0.228
BMI (kg/m^2^)	22.8 (21.2–25.1)	22.3 (19.9–24.4)	23.7 (21.7–25.3)	0.192
SMI (kg/m^2^)	7.0 (6.2–7.5)	6.4 (5.8–7.6)	7.1 (6.2–7.5)	0.302
WBPhA (°)	4.9 (4.5–5.7)	4.8 (4.4–5.2)	5.1 (4.6–5.8)	0.178
Rt HS (kg)	28.5 (25.8–36.0)	28.5 (25.0–31.9)	29.9 (26.4–41.5)	0.388
Lt HS (kg)	29.4 (23.4–35.5)	29.0 (23.1–33.4)	30.4 (23.7–37.0)	0.517
Rt LLS (kg)	26.3 (16.9–42.9)	17.5 (12.3–27.7)	30.4 (23.3–45.8)	0.015
Lt LLS (kg)	25.1 (15.1–41.7)	14.1 (11.7–26.9)	30.6 (21.3–43.7)	0.010
Disease (COPD/ACO/Asthma)	13/4/12	7/1/3	6/3/9	0.282
Smoking history (Cu/Ex/Non)	4/19/6	2/8/1	2/11/5	0.463
%VC (%)	104.1 (83.7–111.8)	94.6 (69.9–106.0)	108.9 (86.7–117.5)	0.137
%FEV_1_ (%)	83.4 (66.2–101.1)	77.7 (48.0–91.6)	94.9 (68.7–106.6)	0.213
FEV_1_/FVC (%)	69.1 (58.6–74.9)	70.4 (52.6–79.7)	66.3 (58.3–74.7)	0.502
6MWT (m)	412.0 (358.8–441.3)	363.5 (310.0–424.0)	422.0 (399.5–481.0)	0.029

BMI, body mass index; SMI, skeletal muscle mass index; WBPhA, whole–body phase angle; Rt, right; Lt, left, HS, handgrip strength; LLS, Lower Limb Strength; ACO, Asthma and COPD Overlap; Cu, current smoker; Ex, ex-smoker; Non, non-smoker; %, predicted; VC, vital capacity; FVC, forced vital capacity; FEV_1_, forced expiratory volume in one second; 6MWT, 6 min walk test. Data are presented as medians (interquartile range) unless otherwise stated. Differences between groups were assessed using the Wilcoxon test. Categorical data were compared using Pearson’s chi-squared test.

**Table 2:** Correlation and *p*-value between physical factors and small RNA with age.

	Lt LLS		Rt LLS		6MWT	
	*r*	*p*	*r*	*p*	*r*	*p*
miR-369-3p	−0.5691	0.0016	−0.5875	0.0010	0.1039	0.5987
piR-23136	−0.5179	0.0048	−0.5791	0.0012	−0.5058	0.0060
piR-33114	0.5177	0.0048	0.5578	0.0020	0.5151	0.0050
piR-23197	−0.4879	0.0084	−0.4631	0.0131	−0.2430	0.2127
piR-32865	0.4703	0.0115	0.5088	0.0057	0.2406	0.2175
miR-125b-5p	−0.4597	0.0138	−0.5575	0.0021	−0.2766	0.1541
let-7b-5p	0.4377	0.0198	0.4253	0.0241	0.3394	0.0772
miR-25-3p	−0.4347	0.0208	−0.3808	0.0456	−0.1455	0.4633
miR-152-3p	−0.4307	0.0221	−0.5597	0.0020	−0.3949	0.0375
piR-32946	−0.4000	0.0349	−0.3935	0.0383	−0.0178	0.9282
miR-615-3p	−0.3781	0.0473	−0.4009	0.0345	−0.1042	0.5976
miR-204-5p	0.3659	0.0555	0.4049	0.0326	0.2458	0.2074
let-7e-5p	0.3227	0.0940	0.2835	0.1438	0.2413	0.2161
piR-28192	−0.2932	0.1299	−0.2970	0.1248	−0.1574	0.4237
piR-33028	−0.2368	0.2250	−0.3196	0.0974	−0.2956	0.1266
piR-33168	0.1450	0.4617	0.1985	0.3112	0.1216	0.5376

Notes: Spearman’s partial correlation coefficients and *p*-values, adjusted for age, were calculated between each physical factor (Lt LLS, Rt LLS, and 6MWT) and each of the 16 selected small RNAs. Rt, right; Lt, left; LLS, Lower Limb Strength; 6MWT, 6 min walk test. Covariates: age. Showing partial correlation analysis.

## Data Availability

The raw RNA-seq data generated in this study are not publicly available due to IRB restrictions regarding participant privacy. However, data are available from the corresponding author upon reasonable request.

## Supplementary Materials

The following supporting information can be downloaded at: https://www.mdpi.com/article/10.3390/biom15121663/s1, Table S1A: The top 50 small RNAs upregulated in the Frail vs. Non-Frail groups; Table S1B: The top 50 small RNAs downregulated in the Frail vs. Non-Frail groups; Table S2: Top 50 significantly differentially expressed small RNAs in the Frail vs. Non-Frail groups; Table S3: Spearman’s rank correlation coefficient and *p*-value of physical factors for small RNA in all patients; Table S4: Top Analysis Ready Molecules in Ingenuity Pathway Analysis; Table S5: Results of Logistic Regression analysis for frailty in the models including small RNA, age, gender; Figure S1: Flowchart of the study patients; Figure S2. CD63 expression in each SEC fraction, showing the original image corresponding to Figure 1B; Figure S3: Expression of EV-derived three candidate miRNAs: miR-125b-5p, miR-369-3p, and miR-615-3p in the Frail vs. Non-Frail groups; Figure S4: Expression of promising EV-derived piRNAs: piR-23136 and piR-33114 in the Frail vs. Non-Frail groups.

## Abbreviations

The following abbreviations are used in this manuscript:

EV	extracellular vesicle
miRNA	microRNA
piRNA	PIWI-interacting RNA
BMI	body mass index
SMI	skeletal muscle mass index
WBPhA	whole–body phase angle
Rt	right
Lt	left
HS	handgrip strength
LLS	lower limb strength
Cu	current smoker
Ex	ex-smoker
Non	non-smoker
%	predicted
VC	vital capacity
FVC	forced vital capacity
FEV_1_	forced expiratory volume in one second
6MWT	6 min walk test
FC	fold change
