# Supplementary material for: Small RNA Profiles of Serum-Derived Extracellular Vesicles in the Comorbid Condition of Frailty and Obstructive Pulmonary Disease: An Observational, Cross-Sectional Study
Source: Biomolecules. 2025 Nov 28;15(12):1663. doi: 10.3390/biom15121663 (PMC12731022; doi:10.3390/biom15121663)
Supplement: Supplementary file 1 [file biomolecules-15-01663-s001.zip › biomolecules-3969834-supplementary.pdf]

biomolecules

MDPI

*Article*

# Small RNA profiles of serum-derived extracellular vesicles in the comorbid condition of frailty and obstructive pulmonary disease

Table S1A. The top 50 small RNAs upregulated in the Frail vs. Non-Frail groups.

| | Descending order | | |
|---|---|---|---|
| | Small RNA | Frail vs Non-Frail Log $_2$ FC | Frail vs Non-Frail T.test (*p-value*) |
| 1 | piR-23136 | 3.721 | 0.0016224 |
| 2 | miR-1-3p | 3.671 | 0.0107790 |
| 3 | miR-206 | 3.149 | 0.0634803 |
| 4 | piR-33028 | 2.955 | 0.0015667 |
| 5 | piR-32912 | 2.668 | 0.0106529 |
| 6 | piR-12319 | 2.509 | 0.0375549 |
| 7 | miR-133a-3p | 2.414 | 0.0886617 |
| 8 | piR-32886 | 2.336 | 0.0260109 |
| 9 | piR-33043 | 2.084 | 0.0286375 |
| 10 | piR-427 | 2.076 | 0.0030716 |
| 11 | piR-27432 | 2.036 | 0.0631187 |
| 12 | piR-23058 | 2.013 | 0.0631230 |
| 13 | piR-12390 | 2.012 | 0.0640578 |
| 14 | piR-1014 | 1.936 | 0.0026759 |
| 15 | miR-190b-5p | 1.914 | 0.1769224 |
| 16 | piR-28096 | 1.891 | 0.0680850 |
| 17 | piR-1424 | 1.891 | 0.1459211 |
| 18 | piR-1080 | 1.847 | 0.0196261 |
| 19 | piR-12584 | 1.841 | 0.1410100 |
| 20 | piR-27491 | 1.833 | 0.0087417 |
| 21 | piR-3711 | 1.828 | 0.0277201 |
| 22 | piR-27488 | 1.788 | 0.0748923 |
| 23 | piR-33025 | 1.754 | 0.0106020 |
| 24 | piR-32994 | 1.750 | 0.1938777 |

| | | | |
|---|---|---|---|
| 25 | piR-1900 | 1.749 | 0.0674635 |
| 26 | miR-184 | 1.748 | 0.2496428 |
| 27 | miR-100-5p | 1.740 | 0.1069431 |
| 28 | miR-450b-5p | 1.685 | 0.1002551 |
| 29 | piR-32952 | 1.675 | 0.0049267 |
| 30 | piR-32953 | 1.673 | 0.1172436 |
| 31 | piR-28061 | 1.672 | 0.1092077 |
| 32 | piR-1079 | 1.659 | 0.0270529 |
| 33 | miR-369-3p | 1.658 | 0.0402504 |
| 34 | piR-18682 | 1.642 | 0.1413753 |
| 35 | piR-27080 | 1.627 | 0.0059180 |
| 36 | piR-414 | 1.617 | 0.1001146 |
| 37 | piR-1638 | 1.593 | 0.1795756 |
| 38 | piR-14633 | 1.554 | 0.2064826 |
| 39 | piR-32946 | 1.550 | 0.0396487 |
| 40 | piR-866 | 1.546 | 0.1655771 |
| 41 | piR-24828 | 1.535 | 0.0217534 |
| 42 | piR-32914 | 1.530 | 0.0143838 |
| 43 | miR-1468-5p | 1.525 | 0.1399143 |
| 44 | piR-1637 | 1.504 | 0.1488877 |
| 45 | piR-28727 | 1.498 | 0.0214126 |
| 46 | piR-29205 | 1.498 | 0.0214126 |
| 47 | miR-125b-5p | 1.496 | 0.0076602 |
| 48 | miR-12136 | 1.486 | 0.1486075 |
| 49 | miR-215-5p | 1.485 | 0.1848980 |
| 50 | miR-615-3p | 1.470 | 0.0200672 |

FC, fold change

Table S1B. The top 50 small RNAs downregulated in the Frail vs. Non-Frail groups.

| | Ascending order | | |
|---|---|---|---|
| | Small RNA | Frail vs Non-Frail<br>$Log_2$ FC | Frail vs Non-Frail<br>T.test (*p-value*) |
| 1 | miR-204-5p | -3.315 | 0.0003141 |
| 2 | miR-431-5p | -3.030 | 0.0018337 |

| | | | |
|---|---|---|---|
| 3 | miR-29c-5p | -2.890 | 0.0011234 |
| 4 | miR-1268a | -2.843 | 0.0026320 |
| 5 | miR-625-3p | -2.842 | 0.0074955 |
| 6 | miR-664a-5p | -2.836 | 0.0024550 |
| 7 | piR-33168 | -2.742 | 0.0176760 |
| 8 | miR-1307-5p | -2.669 | 0.0031942 |
| 9 | miR-132-3p | -2.657 | 0.0059898 |
| 10 | miR-20b-5p | -2.653 | 0.0103085 |
| 11 | miR-4433b-3p | -2.637 | 0.0106377 |
| 12 | miR-326 | -2.580 | 0.0181442 |
| 13 | miR-95-3p | -2.517 | 0.0203605 |
| 14 | miR-625-5p | -2.429 | 0.0075003 |
| 15 | miR-186-5p | -2.411 | 0.0139072 |
| 16 | miR-18a-5p | -2.344 | 0.0344543 |
| 17 | miR-483-3p | -2.318 | 0.0037336 |
| 18 | miR-628-5p | -2.295 | 0.0223977 |
| 19 | miR-199b-5p | -2.282 | 0.0099485 |
| 20 | miR-1301-3p | -2.226 | 0.0278940 |
| 21 | miR-197-3p | -2.194 | 0.0228452 |
| 22 | miR-5187-5p | -2.138 | 0.0247633 |
| 23 | miR-139-5p | -2.080 | 0.0320633 |
| 24 | miR-421 | -2.063 | 0.0255689 |
| 25 | miR-4732-5p | -2.058 | 0.0403314 |
| 26 | miR-224-5p | -2.028 | 0.0257503 |
| 27 | miR-1273h-3p | -2.013 | 0.0054759 |
| 28 | miR-1180-3p | -1.998 | 0.0212182 |
| 29 | miR-26b-3p | -1.975 | 0.0289097 |
| 30 | piR-32865 | -1.968 | 0.0351667 |
| 31 | miR-345-5p | -1.962 | 0.0554505 |
| 32 | miR-339-3p | -1.944 | 0.0538249 |
| 33 | miR-3960 | -1.922 | 0.0315948 |
| 34 | piR-1911 | -1.866 | 0.0345647 |
| 35 | miR-148b-5p | -1.849 | 0.0552552 |
| 36 | miR-335-5p | -1.833 | 0.0158904 |

| | | | |
|---|---|---|---|
| 37 | piR-32166 | -1.813 | 0.0629977 |
| 38 | miR-1908-5p | -1.804 | 0.0385464 |
| 39 | miR-6842-3p | -1.797 | 0.0355922 |
| 40 | piR-20636 | -1.787 | 0.1051470 |
| 41 | miR-1247-5p | -1.758 | 0.0088970 |
| 42 | miR-9-5p | -1.750 | 0.0946294 |
| 43 | piR-28263 | -1.748 | 0.1020170 |
| 44 | piR-32161 | -1.744 | 0.0540268 |
| 45 | miR-331-3p | -1.739 | 0.1067868 |
| 46 | miR-181c-3p | -1.729 | 0.0228976 |
| 47 | miR-425-3p | -1.724 | 0.0935831 |
| 48 | miR-6852-5p | -1.709 | 0.0747648 |
| 49 | miR-320b | -1.696 | 0.0777476 |
| 50 | piR-32237 | -1.688 | 0.1223037 |

FC, fold change

Table S2. Top 50 significantly differentially expressed small RNAs in the Frail vs. Non-Frail groups.

| | small RNA | Frail vs Non-Frail T-test (*p-value*) | Frail vs Non-Frail FDR (*q-value*) | Frail vs Non-Frail $Log_2$ FC |
|---|---|---|---|---|
| 1 | miR-126-3p | 0.0000247 | 0.02527624 | -1.0831790 |
| 2 | miR-204-5p | 0.0003141 | 0.1609945 | -3.3145180 |
| 3 | miR-150-5p | 0.0007387 | 0.2363283 | -1.0467894 |
| 4 | miR-29c-5p | 0.0011234 | 0.2363283 | -2.8902158 |
| 5 | piR-33028 | 0.0015667 | 0.2363283 | 2.9549082 |
| 6 | piR-23136 | 0.0016224 | 0.2363283 | 3.7209271 |
| 7 | miR-431-5p | 0.0018337 | 0.2363283 | -3.0301384 |
| 8 | miR-451a | 0.0020420 | 0.2363283 | 1.2619770 |
| 9 | miR-664a-5p | 0.0024550 | 0.2363283 | -2.8360633 |
| 10 | miR-1268a | 0.0026320 | 0.2363283 | -2.8433787 |
| 11 | piR-1014 | 0.0026759 | 0.2363283 | 1.9364893 |
| 12 | piR-427 | 0.0030716 | 0.2363283 | 2.0757751 |
| 13 | miR-1307-5p | 0.0031942 | 0.2363283 | -2.6689726 |
| 14 | miR-101-3p | 0.0033364 | 0.2363283 | 0.7499056 |
| 15 | miR-483-3p | 0.0037336 | 0.2363283 | -2.3178190 |

| | | | | |
|---|---|---|---|---|
| 16 | miR-21-5p | 0.0038830 | 0.2363283 | 0.9387962 |
| 17 | piR-23020 | 0.0039190 | 0.2363283 | -0.9675473 |
| 18 | piR-32845 | 0.0042657 | 0.2429445 | -1.3421120 |
| 19 | miR-25-3p | 0.0045528 | 0.2456451 | 0.7852845 |
| 20 | piR-32952 | 0.0049267 | 0.2525303 | 1.6745935 |
| 21 | miR-125a-5p | 0.0052816 | 0.2551637 | -0.6230124 |
| 22 | miR-1273h-3p | 0.0054759 | 0.2551637 | -2.0127900 |
| 23 | piR-27080 | 0.0059180 | 0.2558531 | 1.6274801 |
| 24 | miR-132-3p | 0.0059898 | 0.2558531 | -2.6568931 |
| 25 | miR-223-3p | 0.0065464 | 0.2610371 | -0.6726144 |
| 26 | piR-23019 | 0.0066205 | 0.2610371 | -1.2223186 |
| 27 | piR-32852 | 0.0069303 | 0.2617616 | -1.2637676 |
| 28 | miR-625-3p | 0.0074955 | 0.2617616 | -2.8421297 |
| 29 | miR-625-5p | 0.0075003 | 0.2617616 | -2.4286859 |
| 30 | miR-125b-5p | 0.0076602 | 0.2617616 | 1.4961356 |
| 31 | piR-27491 | 0.0087417 | 0.2833353 | 1.8330289 |
| 32 | miR-1247-5p | 0.0088970 | 0.2833353 | -1.7579963 |
| 33 | let-7b-5p | 0.0095153 | 0.2833353 | -0.4446084 |
| 34 | miR-199b-5p | 0.0099485 | 0.2833353 | -2.2817268 |
| 35 | miR-20b-5p | 0.0103085 | 0.2833353 | -2.6525552 |
| 36 | piR-33025 | 0.0106020 | 0.2833353 | 1.7535232 |
| 37 | miR-4433b-3p | 0.0106377 | 0.2833353 | -2.6365722 |
| 38 | piR-32912 | 0.0106529 | 0.2833353 | 2.6677368 |
| 39 | miR-1-3p | 0.0107790 | 0.2833353 | 3.6713507 |
| 40 | let-7e-5p | 0.0115660 | 0.2964211 | -0.5662866 |
| 41 | miR-126-5p | 0.0137301 | 0.3315577 | -0.4841722 |
| 42 | miR-152-3p | 0.0138151 | 0.3315577 | 1.1053271 |
| 43 | miR-186-5p | 0.0139072 | 0.3315577 | -2.4110199 |
| 44 | piR-32914 | 0.0143838 | 0.3351257 | 1.5295726 |
| 45 | piR-23197 | 0.0157678 | 0.3500289 | 1.4185478 |
| 46 | miR-335-5p | 0.0158904 | 0.3500289 | -1.8327734 |
| 47 | piR-33114 | 0.0166769 | 0.3500289 | -0.7984662 |
| 48 | miR-486-5p | 0.0167085 | 0.3500289 | 0.6928680 |
| 49 | miR-92a-3p | 0.0169171 | 0.3500289 | 0.6878196 |

| | | | | |
|---|---|---|---|---|
| 50 | miR-143-3p | 0.0171157 | 0.3500289 | 0.9737999 |

FC, fold change

Table S3. Spearman's rank correlation coefficient and *p*-value of physical factors for small RNA in all patients.

| | Lt LLS | | Rt LLS | | 6MWT | |
|---|---|---|---|---|---|---|
| | ϱ | *p* | ϱ | *p* | ϱ | *p* |
| piR-23136 | -0.5781 | 0.0013 | -0.6139 | 0.0005 | -0.535 | 0.003 |
| let-7b-5p | 0.5696 | 0.0016 | 0.5145 | 0.0051 | 0.402 | 0.034 |
| miR-369-3p | -0.5337 | 0.0034 | -0.5707 | 0.0015 | 0.069 | 0.726 |
| piR-33114 | 0.5310 | 0.0036 | 0.5753 | 0.0014 | 0.531 | 0.004 |
| miR-152-3p | -0.5247 | 0.0041 | -0.6207 | 0.0004 | -0.442 | 0.019 |
| miR-125b-5p | -0.5001 | 0.0067 | -0.5818 | 0.0012 | -0.319 | 0.098 |
| let-7e-5p | 0.4878 | 0.0085 | 0.4034 | 0.0333 | 0.327 | 0.089 |
| miR-615-3p | -0.4801 | 0.0097 | -0.4729 | 0.0110 | -0.177 | 0.366 |
| piR-28192 | -0.4775 | 0.0102 | -0.4168 | 0.0274 | -0.257 | 0.187 |
| piR-32865 | 0.4677 | 0.0121 | 0.5165 | 0.0049 | 0.255 | 0.191 |
| miR-204-5p | 0.4673 | 0.0122 | 0.4764 | 0.0104 | 0.308 | 0.111 |
| piR-23197 | -0.4430 | 0.0182 | -0.4483 | 0.0167 | -0.246 | 0.207 |
| piR-33028 | -0.4318 | 0.0218 | -0.4310 | 0.0220 | -0.364 | 0.057 |
| piR-32946 | -0.4299 | 0.0224 | -0.4253 | 0.0241 | -0.059 | 0.767 |
| piR-33168 | 0.3982 | 0.0358 | 0.3600 | 0.0598 | 0.241 | 0.217 |
| miR-25-3p | -0.3829 | 0.0443 | -0.3574 | 0.0619 | -0.142 | 0.471 |

Notes: Spearman's rank correlation coefficients and p-values were calculated between the top 100 small RNAs that showed significant differences in expression levels between the frail and non-frail groups (as listed in Table S2) and the physical performance measures: Lt LLS, Rt LLS, and 6MWT. We selected, 16 small RNAs that showed a significant correlation with Lt LLS ($p < 0.05$). Lt LLS was prioritized because it showed the largest difference between the frail and non-frail groups in Table 1. Characteristics of the study patients.

Rt, right; Lt, left; LLS, Lower Limb Strength; 6MWT, 6-minute walk test. ϱ is Spearman's rank correlation coefficient.

Table S4. Top Analysis Ready Molecules in Ingenuity Pathway Analysis.

| | $Log_2$ FC | |
|---|---|---|
| miR-1-3p | 3.671 | ↑ |
| miR-369-3p | 1.658 | ↑ |
| miR-125b-5p | 1.496 | ↑ |
| miR-615-3p | 1.470 | ↑ |
| miR-451a | 1.262 | ↑ |
| miR-148a-3p | 1.105 | ↑ |
| miR-143-3p | 0.974 | ↑ |
| miR-21-5p | 0.939 | ↑ |
| miR-16-5p | 0.853 | ↑ |
| miR-92a-3p | 0.785 | ↑ |
| miR-204-5p | -3.315 | ↓ |
| miR-431-5p | -3.030 | ↓ |
| miR-29c-5p | -2.890 | ↓ |
| miR-1268a | -2.843 | ↓ |
| miR-625-3p | -2.842 | ↓ |
| miR-4794 | -2.836 | ↓ |
| miR-1307-5p | -2.669 | ↓ |
| miR-132-3p | -2.657 | ↓ |
| miR-17-5p | -2.653 | ↓ |
| miR-4433b-3 | -2.637 | ↓ |

FC, fold change

Table S5. Results of Logistic Regression analysis for frailty in the models including small RNA, age, gender.

| | Frail<br>OR (95% CI) | *p*-value |
|---|---|---|
| miR-125b-5p | 2.14 (1.07-5.95) | 0.0293 |
| Age | 1.07 (0.95 -1.24) | 0.2693 |
| Gender (males versus females) | 1.87 (0.18-43.56) | 0.6138 |
| miR-615b-3p | 82.49 (0- Inf) | 0.0431 |

| | | |
|---|---|---|
| Age | 1.09 (0.96-1.27) | 0.1987 |
| Gender (males versus females) | 1.76 (0.17-39.90) | 0.6464 |
| miR-369b-3p | 1.41 (0.93-2.34) | 0.1066 |
| Age | 1.08 (0.97-1.25) | 0.1748 |
| Gender (males versus females) | 2.13 (0.22-48.6) | 0.5321 |
| piR-23136 | 1.62 (1.18-2.48) | 0.0020 |
| Age | 1.07 (0.93-1.25) | 0.1591 |
| Gender (males versus females) | 7.97 (0.48-371.48) | 0.3637 |
| piR-33114 | 0.21 (0.03-0.71) | 0.0095 |
| Age | 1.08 (0.95-1.27) | 0.2048 |
| Gender (males versus females) | 7.76 (0.38-809.59) | 0.2387 |

OR, odds ratio; CI, confidence interval; Inf, infinite. ORs are presented per 1-unit increase in each variable.

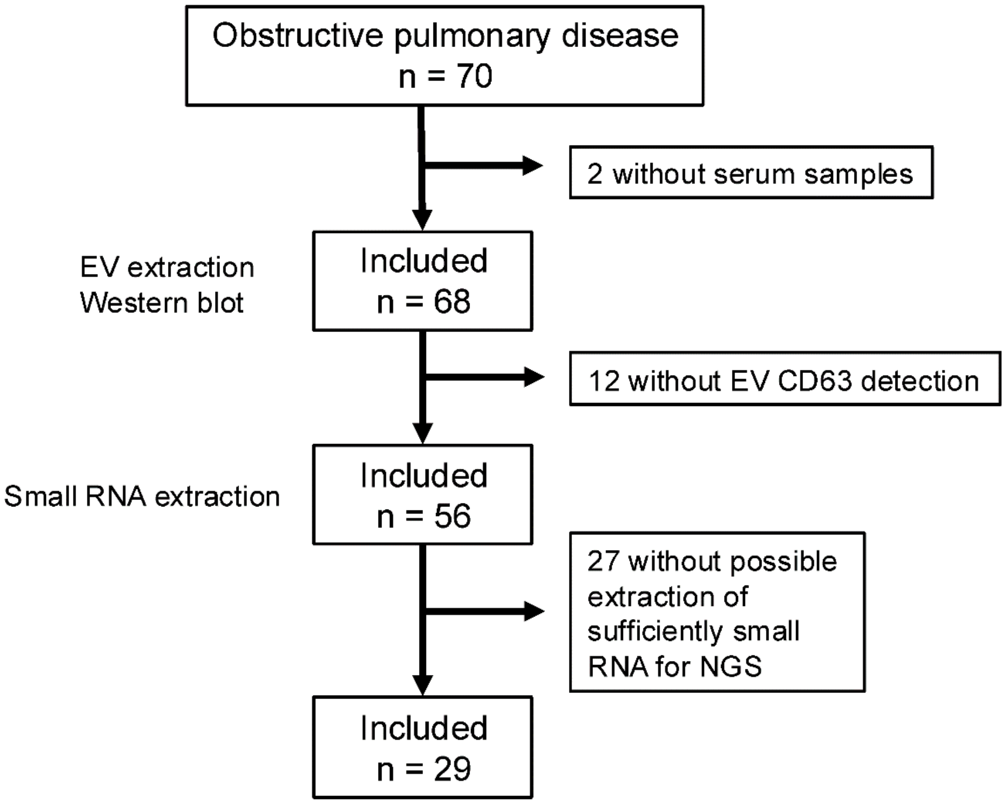


**Figure S1.** Flowchart of the study participants.

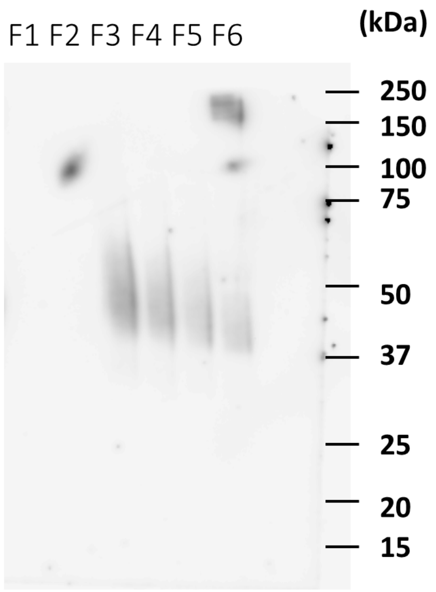


**Figure S2.** CD63 expression in each SEC fraction, showing the original image corresponding to Figure 1B.

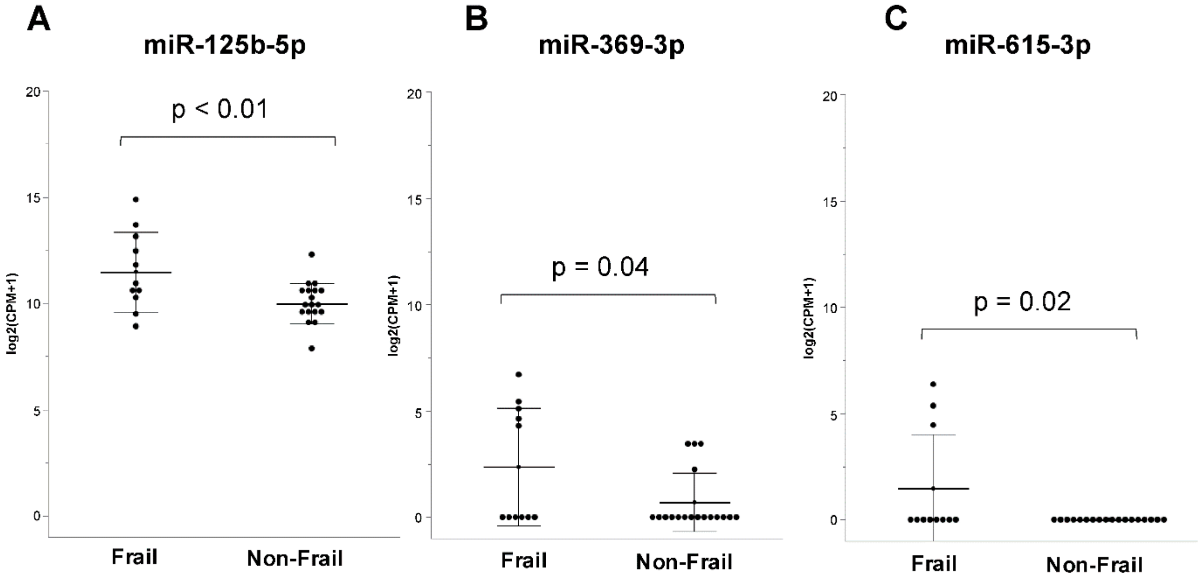


**Figure S3.** Expression of EV-derived three candidate miRNAs: miR-125b-5p, miR-369-3p, and miR-615-3p in the Frail vs. Non-Frail groups.

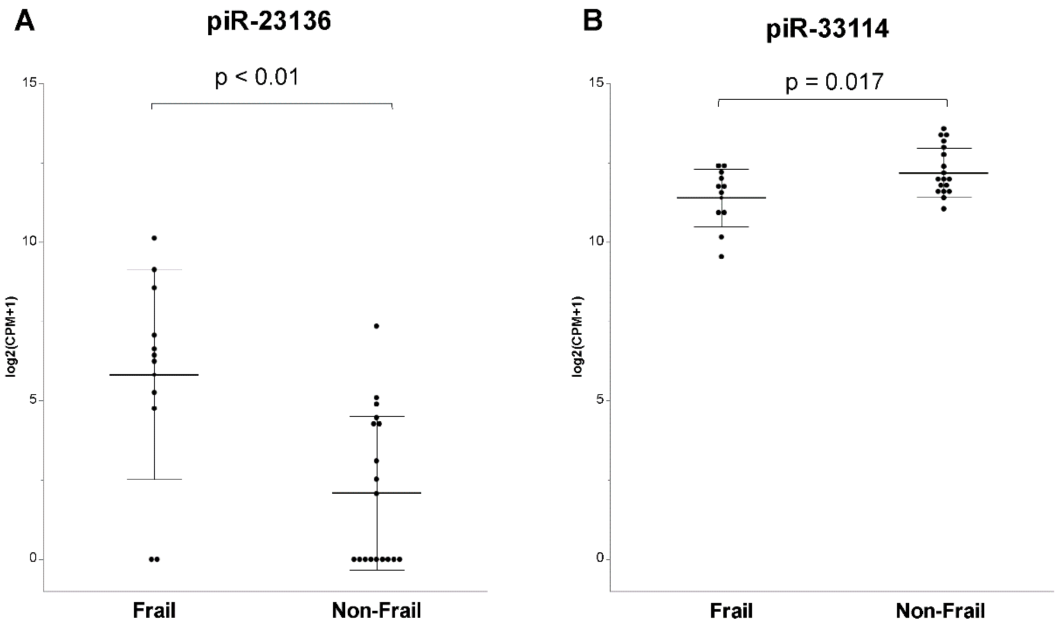


**Figure S4.** Expression of promising EV-derived piRNAs: piR-23136 and piR-33114 in the Frail vs. Non-Frail groups.

**Disclaimer/Publisher's Note:** The statements, opinions and data contained in all publications are solely those of the individual author(s) and contributor(s) and not of MDPI and/or the editor(s). MDPI and/or the editor(s) disclaim responsibility for any injury to people or property resulting from any ideas, methods, instructions or products referred to in the content.
